# In breast cancer subtypes steroid sulfatase (STS) is associated with less aggressive tumour characteristics

**DOI:** 10.1038/s41416-018-0034-9

**Published:** 2018-03-22

**Authors:** Keely M McNamara, Fouzia Guestini, Torill Sauer, Joel Touma, Ida Rashida Bukholm, Jonas C Lindstrøm, Hironobu Sasano, Jürgen Geisler

**Affiliations:** 1 Department of Anatomic Pathology, School of Graduate Medicine, Tohoku University Japan, Sendai, Japan; 2 Department of Pathology, Akershus University Hospital, Lørenskog, Norway; 3 Institute of Clinical Medicine, University of Oslo, Oslo, Norway; 4 Department of Breast- and Endocrine Surgery, Akershus University Hospital, Lørenskog, Norway; 5 Helse Sør-Øst Health Services Research Centre, Akershus University Hospital, Lørenskog, Norway; 6 Department of Oncology, Akershus University Hospital, Lørenskog, Norway

**Keywords:** Breast cancer, Endocrine cancer

## Abstract

**Background:**

The majority of breast cancer cases are steroid dependent neoplasms, with hormonal manipulation of either CYP19/aromatase or oestrogen receptor alpha axis being the most common therapy. Alternate pathways of steroid actions are documented, but their interconnections and correlations to BC subtypes and clinical outcome could be further explored.

**Methods:**

We evaluated selected steroid receptors (Androgen Receptor, Oestrogen Receptor alpha and Beta, Glucocorticoid Receptor) and oestrogen pathways (steroid sulfatase (STS), 17β-hydroxysteroid dehydrogenase 2 (17βHSD2) and aromatase) in a cohort of 139 BC cases from Norway. Using logistic and cox regression analysis, we examined interactions between these and clinical outcomes such as distant metastasis, local relapse and survival.

**Results:**

Our principal finding is an impact of STS expression on the risk for distant metastasis (*p*<0.001) and local relapses (*p* <0.001), HER2 subtype (*p*<0.015), and survival (*p*<0.001). The suggestion of a beneficial effect of alternative oestrogen synthesis pathways was strengthened by inverted, but non-significant findings for 17βHSD2.

**Conclusions:**

Increased intratumoural metabolism of oestrogens through STS is associated with significantly lower incidence of relapse and/or distant metastasis and correspondingly improved prognosis. The enrichment of STS in the HER2 overexpressing subtype is intriguing, especially given the possible role of HER-2 over-expression in endocrine resistance.

## Introduction

Primary breast cancer survival rates have improved dramatically over the last three decades, both in relationship to itself and relative to other cancers.^1^ This improvement has been achieved, at least partially, through the increased effectiveness of targeted endocrine therapies such as aromatase inhibition^2^ and modulation of oestrogen receptor signalling, as well as the development of monoclonal antibodies targeting the HER2/neu receptor.^3^ Despite these manifest improvements in prognosis there are still some areas in breast cancer treatment that remain challenging. These include the identification of more aggressive vs indolent cancers, the treatment of inherently difficult subtypes such as triple negative breast cancer (TNBC; ERα, PR negative, HER2 not overexpressed), de novo and acquired resistance to therapy causing therapeutic failure during adjuvant therapy and, finally, treatment of metastatic disease.

Breast cancer has in general a well-documented dependence on classically female sex steroids, oestrogens, and progesterone. The importance of these hormones in the etiology and maintenance of breast cancer is illustrated in the effectiveness of the therapeutic approaches based on oestrogen depletion or blockade. However, in an attempt to meet the challenges above, the study of the wider range of steroidogenic pathways in the breast is becoming a core component. This wider range of targets encompasses a broader view of pathways that may modulate oestrogen action as well as other classes of steroid molecules such as androgens and glucocorticoids. The overall goal is to explore the cross-talk between the involved steroidal pathways to better understand and overcome, or at least further delay, resistance to endocrine therapy.

**Fig. 1:**
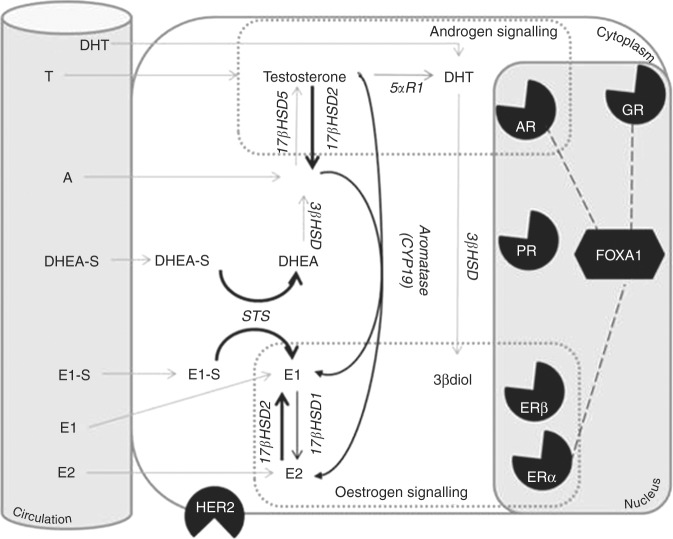
Overview of the steroidogenic pathways thought to be functional in the breast. The classical steroid receptors thought to govern breast cancer prognosis are the oestrogen receptor alpha (ERα) and the Progesterone Receptor (PR). In addition to these the Human Epidermal Growth Factor Receptor 2 (HER2)is also part of the classical panel used to asses breast cancers. This figure demonstrate the extended endocrine environment of the breast with pathways considered in this paper in black, and additional important and potential pathways not studied in grey. This figure is not intended to be a comprehensive diagram of all possible intracrine pathways present in the breast but a guide to the reader of this paper to help orientate them to the significance of the various proteins examined. Circulating precursors such as DHEA-S and E1-S are found in high concentrations in the circulation as are smaller levels of more active steroids such as oestrone (E1), estradiol (E2), Androstenedione (A) and testosterone and cortisol (not shown). Through a series of enzymatic conversions these steroids can be modulated to have greater or lesser activity on a variety of nuclear receptors such as the androgen receptor (AR), oestrogen receptor beta (ERβ) and glucocorticoid receptor (GR) in addition to the classical hormone receptors. Beyond the actions of nuclear receptors the role of cofactors such as FOXA1 and their interactions with hormone receptors are thought to be central to understanding this complex network of interactions

Steroid metabolising enzymes other than aromatase have long been considered potential candidates for breast cancer therapy and important components in the modulation of localised oestrogen levels^4–8^ (Fig. 1). In particular, expression of the STS, 17βHSD1, and 17βHSD2 enzymes have been considered central and important players. 17βHSD1 and 2 have opposite actions modulating the 17 C functional group between a hydroxyl (–OH) and ketone (=O) formation, and thus controlling the relative levels of, e.g., estradiol and estrone.^9,10^ 17βHSD1 catalyses the less potent estrone to the more potent estradiol while 17βHSD2 performs the reverse function. Steroid sulfatase (STS) functions to convert sulfated steroids to their non-sulfated forms (“free steroids”).^9^ This is important as many steroids circulate in their sulfated form in vast excess compared to the non-conjugated forms and thus the presence of STS indicates the ability of the tumour cell to potentially tap a greatly increased reserve of steroids. Previous studies investigating the impact of these enzymes on breast cancers have found that they are influencing BC prognosis, most probably through an alternate oestrogen supply to the breast tissue.^7,11–13^ On the basis of this and other potential therapeutic applications a variety of STS inhibitors have been developed in the last three decades and investigated in preclinical models as potential therapeutic agents in breast cancer,^14,15^ comprehensively reviewed in refs.^16,17^ Results from initial and subsequent clinical trials examining the efficiency of a first generation STS inhibition (STX64/Irosustat) in advanced breast cancer show promising results.^18,19^

In addition, androgens have been a resurgent area of breast cancer research over the last decade. Androgens were originally proposed as agonists in the treatment of breast cancer,^20,21^ however, their use was discontinued due to the efficacy and tolerability of tamoxifen. In the modern era of research into androgen actions in breast cancers, a great deal of focus has recently been placed on their actions in the triple negative subtype,^22^ partially through the appeal of androgen modulation as an effective and available therapeutic treatment for these difficult to treat cancers.^23^ Beyond this the potential of androgen modulation even in ER positive subtypes is once again being considered.^24,25^

Glucocorticoid effects in primary tumours have been a poorly investigated area of breast cancer biology. In the limited studies available, most suggest that the presence of the glucocorticoid receptor in tissue predicts a worse prognosis (Reviewed in refs.^26,27^). However, there is some suggestion that this may be dependent on ER status with worse prognosis observed in ER negative disease but better prognosis observed in ER positive disease.^28^ Overall, the effect of glucocorticoids is thought to be inhibition of both proliferation and apoptosis, the latter being the most concerning in the context of cancer chemotherapy.

Finally, often these pathways are studied in isolation yet they are inherently connected at multiple levels. Androgens serve as an obligate precursor for the local production of oestrogens, the androgen and glucocorticoid receptors are well known to share a DNA binding motif with the FOXA1 transcription factor (Fig. 1) playing an interplay role between these pathways.^29–31^ In this study we sought to analyse the impact of these factors individually but also and importantly in combination across breast cancer tumour subtypes and in relation to clinicopathological factors and outcomes.

## Materials and methods

### Patient cohort

All patients were recruited at the department of breast and endocrine surgery at the Akershus University Hospital, Lørenskog, Norway. Patients gave their written informed consent prior to sampling. The experiments were approved by the Regional Ethics Committee of South-East Norway (Project number: 2014–895). Patients' characteristics are summarised in Table 1. ER, PR, and HER2 status was drawn from the clinical records of patients and was evaluated as follows. Histological samples were fixed in Neutral buffered formalin and paraffin embedded (Table 2). For immunohistochemistry 3 micron thick sections were cut and subsequently stained with the Ventana Benchmark Ultra immunostainer (Ventana Medical Systems, Roche) with iView DAB Detection Kit (Roche) and ultraView Universal Alkaline Phosphatase Red Detection Kit (Roche). The following are the antibodies and criteria used for positivity in determining the ER/PR and HER2 status of the tumour; ER: CONFIRM anti-Oestrogen Receptor (ER) (SP1) Rabbit Monoclonal Primary Antibody (Roche) Positive nuclear staining in >1% of tumour cell nuclei registered as positive irrespective of staining intensity; PR: CONFIRM anti-Progesterone Receptor (PR) (1E2) Rabbit Monoclonal Primary Antibody (Roche).

**Table 1:** Clinicopathological charateristics

Variable	Value (*N*)		
	Whole cohort	Post-menopausal	Pre-menopausal
Age			
Mean	60.5	66.4	41.1
Highest	92	92	51
Lowest	34	49	34
Grade			
1	8 (5.9%)	4 (4.3%)	2 (12.5%)
2	69 (50.8%)	50 (53.7%)	4 (25.0%)
3	59 (43.3%)	39 (41.9%)	10 (62.5%)
Tumour size (T)			
1 (<2 cm)	63 (45.3%)	47 (48.9%)	4 (25.0%)
2 (2–5 cm)	70 (50.4%)	47 (48.9%)	10 (62.5%)
3 (>5 cm)	6 (4.3%)	2 (2.1%)	2 (12.5%)
Nodal spread (N)			
0 (no spread to lymph)	77 (55.8%)	54 (56.8%)	9 (56.2%)
1 (1–3 pos. lymph nodes)	36 (26.1%)	26 (27.3%)	3 (18.75%)
2 (4–9 pos. lymph nodes)	14 (10.1%)	11 (11.5%)	1 (6.2%)
3 (>10 pos. lymph nodes)	11 (8.0%)	4 (4.2%)	3 (18.75)
Metastasis (M)			
No Met	137 (96.6%)	93 (97.8%)	16 (100%)
Mets	2 (1.4%)	2 (2.2%)	0 (0%)
Menopausal status			
Pre-menopausal	16 (11.5%)		
Post-menopausal	96 (69.1%)		
Unknown	27 (19.4%)	Excluded	
BC subtype			
HER2	37 (26.6%)	26 (27.2%)	6 (37.5%)
LUMA	74 (53.2%)	49 (51.0%)	7 (43.7%)
LUMB	11 (7.9%)	8 (8.3%)	0 (0%)
TNBC	17 (12.2%)	13 (13.5%)	3 (18.75%)
Relapse (local and metastatic)			
No	94 (67.7%)	64 (68.1%)	11 (68.7%)
Yes	45 (32.3%)	30 (31.9%)	5 (31.2%)
Relapse (metastatic)			
No	96 (69.1%)	62 (66.7%)	11 (68.7%)
Yes	43 (30.9%)	31 (33.3%)	5 (31.2%)
Oestrogen receptor α			
Negative	36 (25.9%)	26 (27.1%)	5 (31.2%)
Positive	103 (74.1%)	70 (71.9%)	11 (68.7%)
PGR			
Negative	74 (53.2%)	53 (55.2%)	7 (42.7%)
Positive	65 (46.8%)	43 (44.8%)	9 (56.3%)
HER2 over-expression			
No	99 (71.2%)	67 (69.8%)	10 (62.5%)
Yes	40 (28.8%)	29 (30.2%)	6 (37.5%)
STERSULF			
Negative	57 (41.3%)	42 (44.2%)	6 (37.5%)
Positive	81 (58.7%)	53 (55.8%)	10 (62.5%)
Aromatase			
Score 1–4	40 (28.2%)	27 (38.6%)	3 (30%)
Score 5–7	90 (71.2%)	43 (61.4%)	7 (70%)
17βHSD Type 2			
Negative	25 (18.1%)	21 (22.1%)	2 (12.5%)
Positive	113 (81.9%)	74 (77.9%)	14 (87.5%)
ERβ1			
<150 H Score	65 (46.7%)	47 (49.0%)	7 (43.7%)
>150 H score	74 (53.2%)	49 (51.0%)	9 (56.3%)
AR			
<10%	18 (13.3%)	12 (13.0%)	1 (6.25%)
≥10%	117 (86.7%)	80 (87.0%)	15 (93.75%)
GR			
<10%	64 (48.5%)	41 (45.1%)	11 (73.3%)
≥10%	68 (51.5%)	50 (54.1%)	4 (26.6%)
FOXA1			
<10%	0 (0%)	0 (0%)	0 (0%)
≥10%	132 (100%	90 (100%)	16 (100%)
KI67			
<15	31 (22.6%)	21 (22.3%)	2 (12.5%)
15–30	37 (27.0%)	26 (27.7%)	2 (12.5%)
>30	69 (50.4%)	47 (50%)	12 (75.0%)

Clinicopathological and histological characteristics of the cohort

Positive nuclear staining in >10% of tumour cell nuclei registered as positive irrespective of staining intensity. HER-2 antibody: PATHWAY anti-HER-2/neu (4B5) Rabbit Monoclonal Primary Antibody (Roche) Membrane staining evaluated as follows: no staining (=0), weak to moderate incomplete staining (=1+), moderate complete membrane staining in >10% of tumour cells (=2+) and marked/intense membrane staining in >10% of tumour cells (=3+). 0 and 1 + registered as HER-2 negative case 2+inconclusive, assessed by dual SISH (Roche). 3+ registered as positive for HER-2. INFORM HER2 Dual ISH DNA Probe Cocktail Assay and ultraVIEW SISH Detection Kit (Roche) Dual SISH with silver stained HER-2 signals and red CEP (centromere) 17 signals. A ratio gene signal number/CEP17 signal number >2.0 registers as HER-2 gen amplified. Sufficient clinical material to perform analysis of steroidogenic enzymes was available from 139 patients. The mean age of the study population was 59 years (range: 34–93 years).

### Immunohistochemistry

Immunohistochemistry (IHC) for AR, GR, CYP19 (aromatase), 17βHSD2, STS, FOXA1, and ERβ was performed, as previously described.^12,32–35^ In brief, the following primary antibodies and conditions were employed; (Ki67 (MIB-1) DAKO 1:100; AR (AR441)DAKO 1:50; GR, (D6H2L)Cell Signalling technologies 1:400; AROM (677), Novartis, 1:500; 17βHSD2, Proteintech, 1:200; STS, (KW1049) Kyowa medix, 1:100; FOXA1 (3C1) ABCAM 1:200, ERβ1 1:1000 (Genetex, 14C8)). Heat based antigen retrieval using 10 mM citric acid buffer (ph6.0) was performed for AR, GR, ERβ, FOXA1, Ki67, STS, aromatase and 17βHSD2. In all cases the Nichirei staining system based upon streptavidin peroxidase conjugation was used for the visualisation of primary antibody binding. The robustness and reproducibility of the method was confirmed by the inclusion of a verified positive control tissue with each IHC run.

The slides were independently evaluated by at least two authors for each stain (KMM, FG) blinded to patients clinical outcomes. Nuclear stains were quantified using the H-score. This gives a measure of intensity and prevalence along a scale of 0–300 on the basis of 5 hot spots in the tissue and the labelling index was used for Ki67. The evaluation of CYP19/aromatase was performed by assessing the approximate percentage of cells staining (proportion score) and classifying the level into four groups: 0 = <1%, 1 = 1–25%, 2 = 26–50%, and 3 = >50% immuno-positive cells, and the relative intensity of immune-positive cells was classified as follows: 0 = no immune-reactivity, 1 = weak, 2 = moderate and 3 = strong immune-reactivity. The total score was the addition of the proportion score and the relative immune-intensity score.^36^ The staining of the other enzymes was assessed across the whole carcinoma section and categorised into one of three groups: no staining (0), <50% staining (1), >50% staining (2)^12,33–35^

### Statistical analysis

Raw data were examined for distribution. Since most of the variables to be considered are categorical, the relationships between them are presented using cross-tables with raw numbers. The STS and 17βHSD2 variables are scored in 3 categories (Negative, 1–50%, greater than 50%) but for the purpose of analysis were transformed into a dichotomous variable, with “negative” coded as negative, and positive otherwise. Ki-67 is a continuous variable and was treated as such in the statistical analyses, but for tabulation purposes it is divided into three ranges: smaller than 15%, 15–30%, and greater than 30%.

Univariate logistic regression modeling was used for statistical inference of the relationship between relapse and metastasis and the enzymes and nuclear receptors. Analysis of variance (ANOVA) and logistic regression was used to test association between endocrine therapy and BC subtype against enzymes and nuclear receptors.

The effect of our endocrine markers on survival from the time of surgery was analysed using Cox regression including the age of the patient as a covariate. The survival time was visualised using Kaplan–Meier curves.

As many tests were performed, it is worthwhile to keep in mind the potential effects of multiple hypothesis testing. As such in the text we have given the actual *P* value rather than using a cut-off point of *p* > 0.05. As a rule of thumb we used the more stringent *p* < 0.005 for considering an association to be statistically significant.

All analyses were done in R version 3.2.0.^37^

## Results

### Localisation, distribution and correlations of steroidogenic markers

**Fig. 2:**
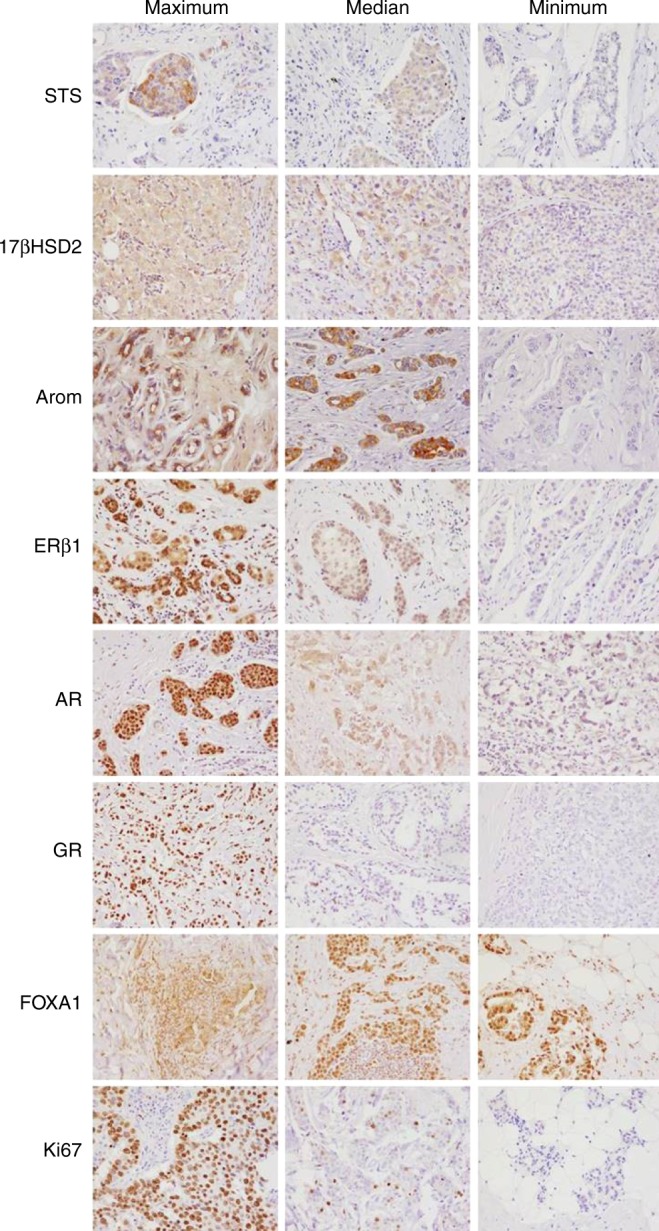
Representative IHC images of immunohistochemical stains in breast cancer samples. For each stain we chose the maximal, median and minimal values of the stain and have shown the representative images (×200 magnification). Note in most cases the epithelial location of the staining. While not illustrated here is should be noted that over and entire section of cancer tissue some of these stains were heterogeneous thus the possibility of steroid expressing subpopulations within the one tumour should not be ruled out. At present however there are few scoring approaches to adequately asses this issue and as such it is not dealt with in this manuscript

Patient cohort characteristics alongside IHC marker expression are summarised in Tables 1 and 2, respectively. As can be seen in the representative images shown in Fig. 2 the most marked immunoreactivity of all markers studied was in the carcinoma cells. AR and GR were observed in a predominantly nuclear localisation while aromatase, STS and 17βHSD2 were mostly cytoplasmic. The proportion of patients that were positive for any stain varied. Correlations between these various markers were observable but relatively weak (below *r* = 0.03, Supplementary Data 1). AR was strongly correlated with GR, FOXA1, ERβ and aromatase; ERβ was strongly correlated with AR, Aromatase and FOXA1; and GR was correlated with STS. FOXA1 was inversely correlated with Ki67 with a similar trend observable for ERβ1. When using a Bonferroni correction to control for multiple comparisons, the only effect that remained below *p* < 0.005 was the association between ERβ1 and aromatase score (*p* = 0.001).

**Table 2:** Breast cancer subtype and marker expression

	Luminal A	Luminal B	HER2	TNBC	*P* value
Oestrogen receptor α					
Positive	74	11	18	0	—
Negative	0	0	19	17	
Progesterone receptor					
Positive	49	6	10	0	—
Negative	25	5	27	17	
STS					
Positive	39	4	28	10	0.0274
Negative	35	7	8	7	
17βHSD2					
Positive	60	9	30	14	0.9934
Negative	14	2	6	3	
Aromatase					
Score 1–4	20	3	10	7	0.69
Score 5–7	54	8	27	10	
Oestrogen receptor β1					
Average±SD	183.8 ± 76.8	147 ± 74.6	164 ± 80.9	126.3 ± 85.9	0.041
Range	21.3–300.5	75.6–264	8.5–313	14–264	
Androgen receptor					
Positive	62	10	35	10	0.0119
Negative	8	1	2	7	
Glucocorticoid receptor					
Positive	40	4	15	9	0.2898
Negative	30	7	21	6	
Ki67					
<15%	25	1	4	1	<0.001
15–30%	24	5	5	3	
>30%	24	5	17	13	

Correlation of nuclear receptors and steroidogenic enzymes with breast cancer subtype

For the nuclear receptors and transcription factor it was necessary for some analysis to create dichotomised values (positive/negative). This was based on a cut-off of 10% labelling index. 74% of cases were positive for ERβ, 86% of cases were considered positive for AR and 51% positive for GR. An additional marker, the important nuclear transcription factor FOXA1 was also included in the analysis. However, 100% of the samples demonstrated immunoreactivity in this cohort and thus it was not possible to dichotomise the cohort into positive and negative values. For the enzymes, dichotomised as described in methods above, a majority of cases were positive to some degree for aromatase, STS and 17βHSD2 (Table 1).

### Relationship between clinicopathological factors, breast cancer subtype and marker expression

In evaluating relationships between clinicopathological characteristics and breast cancer subtypes we found that there was an association between subtype and proliferation with Ki67 levels being lower in the luminal A subtype compared to HER2-positive (*p* < 0.001) and TNBC (*p* < 0.001), as expected. Moreover, luminal A cancers had the highest rates of PR positivity and were statistically more likely to be PR positive compared to HER2 subtype (*p* < 0.001), as expected (Table 2). Interestingly menopausal status seemed to not strongly affect marker expression (Supplementary Table 1) with GR being the only protein suggestive of being linked to menopausal status (*p* = 0.051, Odds ratio 3.35, increased in post-menopausal patients)

**Table 3:** Regression analysis

	Relapse	No relapse	Regression coefficient	Odds ratio	*P* value
**Local relapse**					
Oestrogen receptor α					
Positive	34	69	0.1132	1.12	0.787
Negative	11	25			
Progesterone receptor					
Positive	20	45	−0.138	0.87	0.705
Negative	25	49			
HER2					
Positive	13	27	0.008	1.008	0.984
Negative	32	67			
STS					
Positive	16	65	−1.367	0.255	*<0.001*
Negative	28	29			
17βHSD2					
Positive	41	72	1.429	4.176	0.027
Negative	3	22			
Androgen receptor					
Positive	40	77	0.955	2.59	0.149
Negative	3	15			
Glucocorticoid receptor					
Positive	23	45	0.346	1.413	0.365
Negative	17	47			
Aromatase					
Cont.			0.039	1.039	0.802
Oestrogen receptor β					
Cont.			−0.002	0.998	0.304
Ki67					
Cont.			−0.003	0.99	0.782
**Distal relapse**					
Oestrogen receptor α					
Positive	29	72	−0.034	0.9667	0.938
Negative	10	24			
Progesterone receptor					
Positive	19	46	0.03209	1.032	0.932
Negative	20	50			
HER2					
Positive	13	25	0.3507	1.42	0.394
Negative	25	71			
STS					
Positive	11	68	−1.7852	0.168	*<0.001*
Negative	27	28			
17βHSD2					
Positive	35	74	1.244	3.468	0.055
Negative	3	22			
Androgen receptor					
Positive	34	81	0.598	1.819	0.374
Negative	3	13			
Glucocorticoid receptor					
Positive	21	45	0.5647	1.759	0.167
Negative	13	49			
Aromatase					
Cont.			−0.065	0.937	0.680
Oestrogen receptor β					
Cont.			−0.001	0.999	0.662
Ki67					
Cont.			0.00342	1.033	0.756

Relationships between nuclear receptors, steroidogenic enzymes, and clinical outcome. Samples with a P value falling below 0.05 are given in italics

When examining the relationship between the steroidogenic proteins and breast cancer subtypes via regression analysis, only three patterns were apparent. First, AR expression (H Score) score varied between the TNBC subtype and others (*p* = 0.01) with significantly lower levels in TNBC compared to the Luminal A (*p* = 0.0025) and HER2 subgroupings (*p* = 0.0341). Second, STS expression (dichotomised value) also varied between the subtypes (*p* = 0.027) with lower rates in the luminal groupings compared to the HER2 subgrouping (*p* < 0.015). Finally, ERβ expression (H Score) was associated with significant differences across subtypes (*p* = 0.041) with highest levels in the luminal A subgrouping and the lowest levels in the TNBC grouping and differences in ERβ expression between the luminal A and TNBC groupings (*p* = 0.0077).

### Relationship between markers and recurrence

The relationships between the markers we examined and local recurrence and distal metastasis are given in Tables 3. The marker most strongly associated with recurrence or distal metastasis was STS. Positive STS expression in a tumour correlated with a significantly lower rate of local recurrence or distal metastasis (OR = 0.25, *p* < 0.001; OR = 0.17, *p* < 0.001). 17βHSD2 exhibited a weaker effect in the opposite direction, with positive expression associating with increased rates of local or distal recurrence (OR = 4.17, *p* = 0.03; OR = 3.47, *p* = 0.055). Descriptive statistics regarding the expression of markers in the primary tumour and eventual metastatic site(s) of recurrence are given in Supplementary table 1. Unfortunately, the comparably small numbers for each individual metastatic site precluded us from doing extensive statistical analysis on the correlation between expression and relapse site.

### Relationship between markers and survival

We saw no significant effects of any of our established clinico-pathological parameters (BC subtype, ER, PR, HER2, Ki67) on survival. In the steroidogenic parameters examined, only STS expression was associated with a significant survival benefit (HR = 0.27, *p* < 0.001). This effect was not subtype dependent. GR also impacted survival (Cox proportional hazard, adjusted for age. (HR = 1.006, *p* = 0.038). No other markers were associated with strong survival effects although an increased risk for relapses/metastasis was observed for elevated 17βHSD2 activity (HR = 2.1, *p* = 0.16).

### Interactions with therapeutic treatments (endocrine and chemotherapy)

**Fig. 3:**
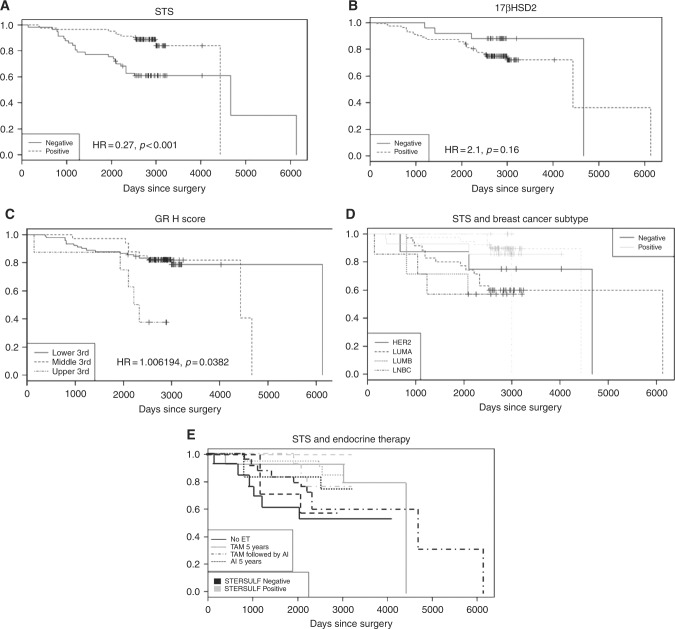
The impact of steroidogenic proteins on overall survival. We detected an effect of STS (**a**), 17βHSD2 (**b**), and GR (**c**) expression on overall survival rates with high levels of STS being associated with longer survival while high levels of 17βHSD2 and GR were associated with shorter survival. Survival analysis examining the interactions of STS expression with breast cancer subtype (**d**) and endocrine therapy (**e**) revealed that the survival benefit associated with STS expression was not confined to one breast cancer subtype or related to a specific endocrine intervention

In an analysis of survival and endocrine treatment no significant differences between endocrine treatment were observed. Given that one of the main presumptive functions of STS is facilitating the supply of oestrogens to the carcinoma from otherwise unavailable circulating steroids, it is interesting to test if there is an interaction between endocrine manipulations and the survival benefit shown by STS expression. In this analysis we did not see any interactions between endocrine treatment and STS effects on survival (Fig. 3), with the outcome of each treatment being affected by STS expression. Likewise there was no correlation between aromatase expression, endocrine treatment and survival outcomes.

### The impact of ERα positivity within the HER2 overexpressed subtype

Given the role of STS in generating oestrogens and the possibility of an ERα positive sub-population in the HER2 overexpressing carcinomas, we tested the impact of ERα expression on STS and aromatase in this grouping. There were no differences in levels of aromatase expression dependent on ERα expression in the HER2 subtype. There was however, an inverse association between ERα expression and STS expression with ERα positive cases being less likely to express STS than ERα negative cases (*p* = 0.039, Regression co-efficient −1.779, Odds ratio 0.16).

### Principal component analysis between variables

Using principle components analysis we examined the relationship between the variables (Supplementary Figure 1). While the data did not reveal any patterns that characterise one subtype over another, it did demonstrate the independence between the set of data.

## Discussion

In this study of the interactions between clinicopathological factors and the extended intracrine tumour environment in a Norwegian breast cancer cohort, our principle finding was a strong protective effect of STS expression on local and distal recurrence as well as improved overall survival. In combination with the inverse trends seen for 17βHSD2 for the same factors, our data seems to suggest that localised synthesis of potent oestrogens by alternative steroid pathways may be protective in breast cancer and/or that depletion of oestrogens via increased metabolism of oestrone may create adverse conditions for cancer cells. A second interaction observed was the strong correlation of HER2 overexpressing carcinomas and STS expression, although HER2 expression appeared to be associated with increased rates of local and distal relapse. One note of caution should be raised in the interpretation of these findings; while we examined the protein expression of STS in the tissues we have not evidence regarding its correlation of STS activity. We are unable to validate if this increased expression indicated increased activity of STS due to the nature of the experimental samples we are working with and such experiments would be an essential future step in unravelling the correlations we have seen in this study. The same cautionary note applies to the subsequent discussion regarding the significance of nuclear receptor expression in the tissues without any information regarding nuclear receptor activity.

Our finding regarding levels of STS expression in breast carcinomas confirms previous reports,^8,12^ while its association with HER2 has also been previously suggested.^7,38^ However, our findings regarding the effects of STS on survival are contradictory to previous reports in the literature, including those from our own laboratory. Previous finding have suggested that STS is increased in malignancy,^7^ and expressed and functional at high levels in invasive cancers.^7,8,11–13,39^ Virtually all previous studies have found that STS expression is associated with worse outcomes and increased recurrence^7,11–13^ albeit with a few dissenting findings.^39^

In reconciling this data with our finding in the present study, we offer a couple of points of note. Firstly, previous studies that rely on mRNA as a surrogate of protein expression^7,11,13^ may not truly reflect the levels of protein or enzymatic activity of STS.^40^ Secondly, the previous studies have focused on specific subtypes of carcinomas, (e.g., luminal A^8^ while the current study investigated a wider range of carcinomas, including the HER2 overexpressing type and TNBC. A final explanation may be that previous studies have predominantly been done in cohorts where the principle ethnicity is Japanese. While it seems unlikely that such drastic differences could exist, effects of ethnicity may not be completely ruled out. These explanations do not reconcile all the difference in the data and await further study in larger cohorts at the protein and ideally enzymatic level.

In meditating on the potential significance of the protective effect of STS on outcomes there are a number of possible explanations to consider. It is interesting to speculate that after the reduction in tumour burden following surgical excision, the presence of local oestrogens may help to maintain residual cells in a luminal-like differentiated state.^41^ This maintenance of differentiation may thus prevent tumour cell senescence and, through the regulation of proliferation, make them more vulnerable to chemotherapy while simultaneously, through actions in EMT, make them less likely to become locally or distally invasive. Established relationships between HER2 actions and stemness make this a fascinating possibility.^42^ Previous research has suggested that ERβ may be present in the HER2 subtype^43^ and that ERβ expression is associated with STS.^38^ While there is not complete consensus on the role or nature of ERβ actions in breast carcinomas (reviewed in^5^ which may be partially due to issues with antibody choice and validation^44,45^ many studies as well as a recent meta-analysis, do suggest it has protective roles.^46^ Thus, local provision of oestrogens or androgens through the DHEA-DHT-3βdiol pathway,^47^ by STS could be protective in this manner. An alternate potential explanation is that the protective actions of STS may not occur exclusively through its actions on oestrogens but through actions on other steroid classes such as androgens (DHEA-S)^48^ or, as observed for other steroidogenic enzymes,^49^ other molecular substrates altogether.

The implications of our findings on the potential of STS inhibition as a therapeutic approach in breast cancers are also problematic. To date preclinical and clinical studies examining STS inhibition in ER positive breast cancers have shown beneficial outcomes; reduced tumour growth and proliferation in pre-clinical models^14,15,50^ and reduced proliferation, steroid levels and prolonged patient survival in clinical trials.^18,19,51,52^ Limited tests have also been done in the preclinical setting for ER negative breast cancers (MDA-MB-231 cell line, TNBC subtype) with similar promising findings.^53^ To the best of our knowledge no tests have been carried out in HER2 models or patients. The best explanation that we can offer to resolve our findings of STS expression being associated with beneficial survival outcomes across breast cancer subtypes, yet STS inhibition being beneficial as detailed above is similar to the explanation offered for the actions of oestrogen inhibition in ERα positive breast cancer or androgen inhibition in AR positive ERα negative breast cancer. This is while the expression of the protein (in this case STS) identifies a cancer with a less aggressive phenotype, thus better survival (e.g., better survival in ERα positive breast cancer vs negative, e.g., Viale et al., ^54^ better survival in AR positive TNBC vs negative^55^ inhibition of that protein may cause growth arrest and thus be beneficial in the long term to patient outcomes. While much of this is speculative, it highlights the importance of further investigation into STS actions and roles in breast cancer to clarify these and other issues.

Beyond the effect on survival observed with STS and 17βHSD2, other important expressions and correlations were observed. Firstly aromatase and ERβ expression were correlated suggesting an interaction between the expressions of these two proteins. While this is logically intuitive there is little data previously reporting this relationship in breast cancers. Given the very different transcriptional program enacted by ERα and ERβ (reviewed in^5^ it is potentially significant for the underlying biology to consider that the impact of aromatase may differ depending on the dominant oestrogen receptor present in the individual carcinoma. Secondly, the data presented in this paper also demonstrated widespread expression of both the AR and GR across breast cancer subtypes. In line with previous reports, AR expression was reduced in the TNBC subtype, albeit with our data at the upper range of what has previously been reported. It is important to note that a subset of TNBC cases retain AR expression both in our dataset and in the literature, and this may be of clinical significance (reviewed in.^23,56^ GR expression has likewise previously been shown to be expressed at comparative levels in breast cancers.^57^ Although non-significant, the sign of both AR and GR regression coefficients suggests a correlation with worse outcome which is consistent with some but not all reports in the literature. While the data from this study did not provide strong evidence as to their biological roles in carcinomas, previous studies have suggested their potential importance to breast cancer biology (reviewed in.^5,27^ Their presence in the tumour across subtypes in this study is of note and should be factored in future investigations.

## Conclusions

The data presented in this paper highlights the presence and complexity of the extended endocrine/intracrine environment of breast cancers. This extended endocrine/intracrine environment is demonstrated by the presence of an expanded panel of nuclear receptors (AR, GR, and ERβ1), as well as the multitude of steroid metabolising enzymes that can interconvert steroid already present in the tumour or make additional pools of circulating steroid available to the tumour. The most striking finding was the existence of a beneficial effect of STS expression in the primary tumour on both local and distal recurrences and on survival. Less striking, but no less important is the data demonstrating that this extended endocrine/intracrine environment exists across breast cancer subtypes including the HER2-positive and TNBC designations. This further understanding may, in the long run, form a keystone in optimising the endocrine treatment of human breast cancer.

## Electronic supplementary material

Supplementry Figures
