# Supplementary material for: In breast cancer subtypes steroid sulfatase (STS) is associated with less aggressive tumour characteristics
Source: Br J Cancer. 2018 Mar 22;118(9):1208–16. doi: 10.1038/s41416-018-0034-9 (PMC5943586; doi:10.1038/s41416-018-0034-9)
Supplement: Supplementary file 1 — Supplementry Figures [file 41416_2018_34_MOESM1_ESM.pdf]

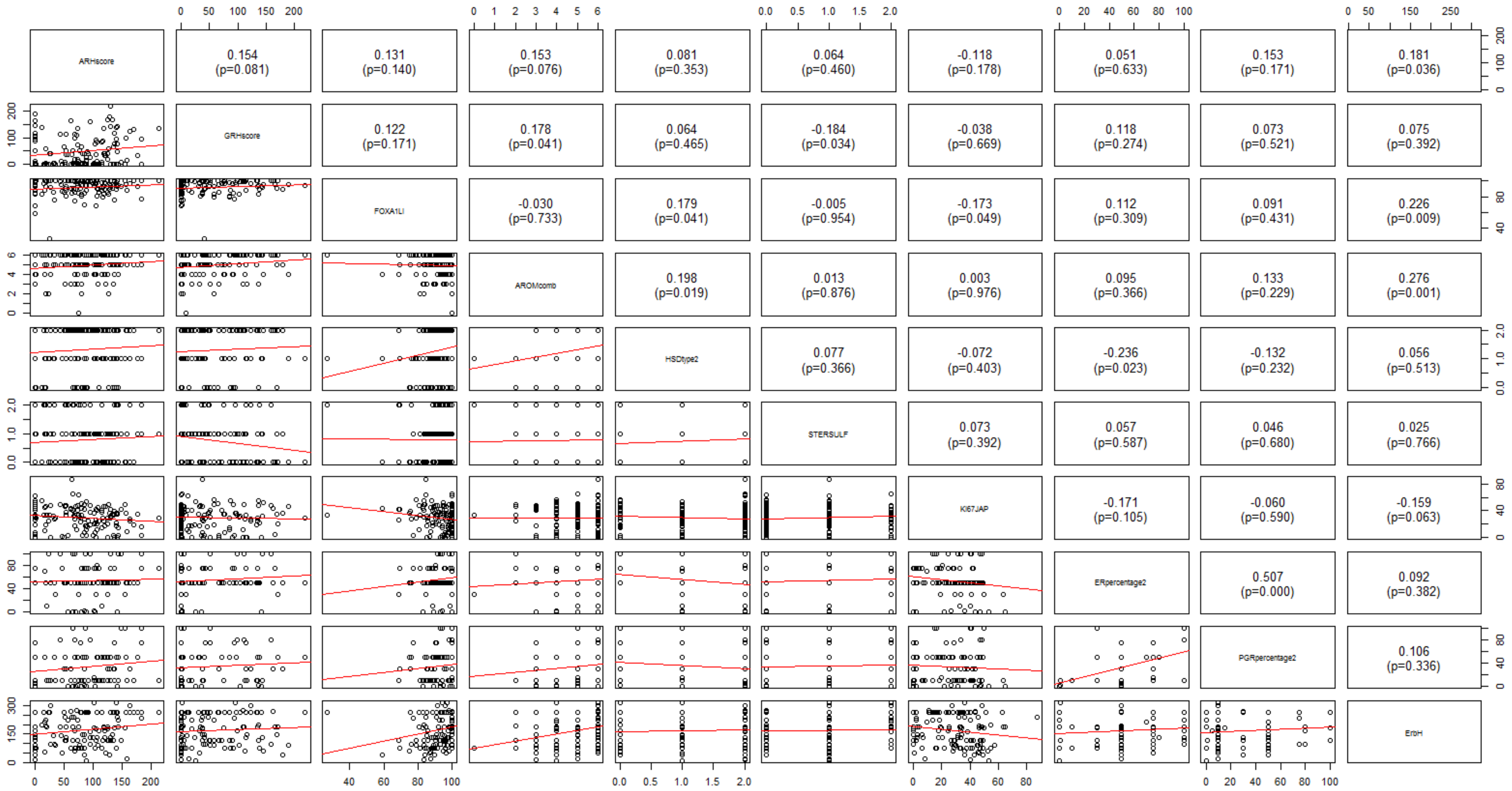

Supplementary Figure 1 Raw correlation data.

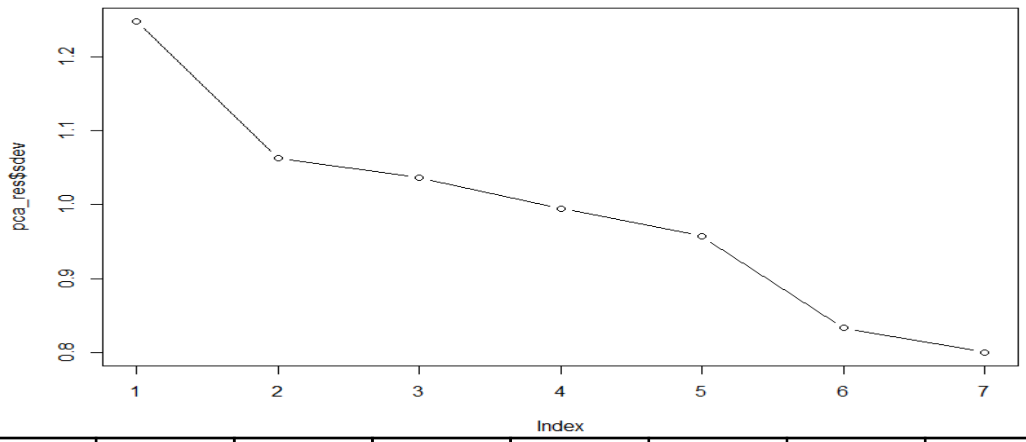

	PC1	PC2	PC3	PC4	PC5	PC6	PC7
Standard deviation	1.2478	1.0628	1.0366	0.9946	0.9572	0.83270	0.80004
Proportion of Variance	0.2224	0.1614	0.1535	0.1413	0.1309	0.09906	0.09144
Cumulative Proportion	0.2224	0.3838	0.5373	0.6786	0.8095	0.90856	1.00000

	PC1	PC2	PC3	PC4	PC5	PC6	PC7
ARHscore	-0,406	-0,128	0,150	0,652	0,321	-0,515	-0,049
GRHscore	-0,504	0,415	-0,052	0,022	0,329	0,464	0,497
FOXA1LI	-0,340	-0,411	-0,540	-0,186	0,328	0,227	-0,480
AROMcomb	-0,441	0,271	0,518	-0,105	-0,293	0,171	-0,581
HSDtype2	-0,357	-0,347	0,264	-0,646	0,023	-0,391	0,334
STERSULF	0,199	-0,551	0,554	0,155	0,269	0,499	0,069
KI67JAP	0,323	0,381	0,197	-0,295	0,723	-0,189	-0,258

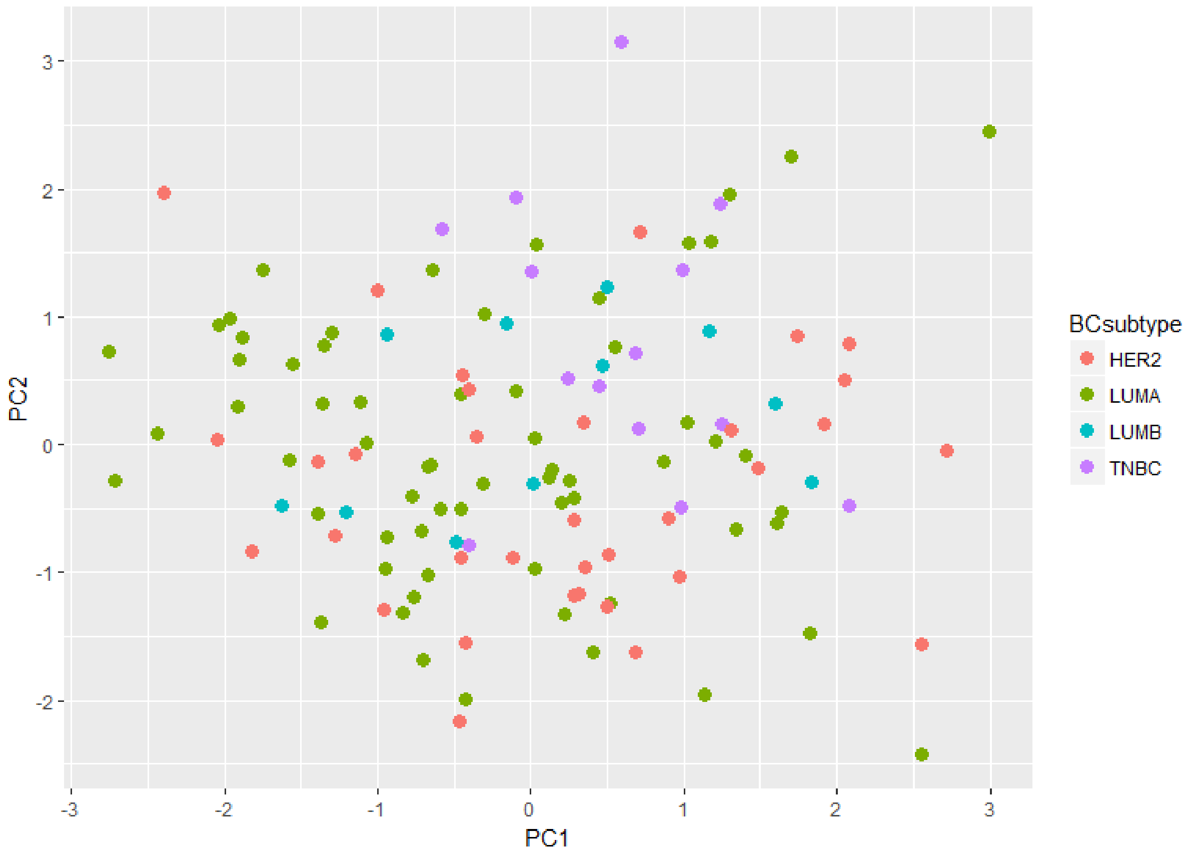

Supplementary Figure 2 Principal component analysis of the dataset

	estimate	odds_ratio	p.value
ER	0.20	1.224	0.73
PGR	-0.46	0.631	0.40
AR	-0.81	0.444	0.45
HER2	-0.33	0.721	0.56
GR	1.21	3.354	0.05
STS	-0.28	0.757	0.62
17βHSD2	-0.69	0.503	0.39
ERβ1	0.00	0.998	0.63
Aromatase	-0.09	0.914	0.72

Supplementary Table 1 Correlations between markers and menopausal status.

Metastatic site	Overall				ERa Status								PGR								HER2 over expression							
					Negative				Positive				Negative				Positive				Negative				Positive			
	no metastasis, %, N		Metastasis %, N		no metastasis, %, N		Metastasis %, N		no metastasis, %, N		Metastasis %, N		no metastasis, %, N		Metastasis %, N		no metastasis, %, N		Metastasis %, N		no metastasis, %, N		Metastasis %, N		no metastasis, %, N		Metastasis %, N	
Bone	86%	119	14%	20	100%	36	0%	0	81%	83	19%	20	92%	68	8%	6	78%	51	22%	14	85%	84	15%	15	88%	35	13%	5
Liver	87%	121	13%	18	97%	35	3%	1	83%	86	17%	17	89%	66	11%	8	85%	55	15%	10	89%	88	11%	11	83%	33	18%	7
Lymph Nodes	92%	128	8%	11	92%	33	8%	3	92%	95	8%	8	92%	68	8%	6	92%	60	8%	5	92%	91	8%	8	93%	37	8%	3
Skin	92%	128	8%	11	86%	31	14%	5	94%	97	6%	6	92%	68	8%	6	92%	60	8%	5	93%	92	7%	7	90%	36	10%	4
Brain	94%	130	6%	9	97%	35	3%	1	92%	95	8%	8	91%	67	9%	7	97%	63	3%	2	94%	93	6%	6	93%	37	8%	3
Lung	94%	130	6%	9	94%	34	6%	2	93%	96	7%	7	89%	66	11%	8	98%	64	2%	1	94%	93	6%	6	93%	37	8%	3
Colon	97%	135	3%	4	92%	33	8%	3	99%	102	1%	1	96%	71	4%	3	98%	64	2%	1	99%	98	1%	1	93%	37	8%	3
Pleura	97%	135	3%	4	94%	34	6%	2	98%	101	2%	2	97%	72	3%	2	97%	63	3%	2	99%	98	1%	1	93%	37	8%	3
Bone Marrow	99%	138	1%	1	100%	36	0%	0	99%	102	1%	1	99%	73	1%	1	100%	65	0%	0	99%	98	1%	1	100%	40	0%	0
Spleen	99%	138	1%	1	100%	36	0%	0	99%	102	1%	1	99%	73	1%	1	100%	65	0%	0	99%	98	1%	1	100%	40	0%	0

	Overall				AR								GR								ERβ1							
					Negative				Positive				Negative				Positive				<150				>150			
	no metastasis, %, N		Metastasis %, N		no metastasis, %, N		Metastasis %, N		no metastasis, %, N		Metastasis %, N		no metastasis, %, N		Metastasis %, N		no metastasis, %, N		Metastasis %, N		no metastasis, %, N		Metastasis %, N		no metastasis, %, N		Metastasis %, N	
Bone	86%	119	14%	20	94%	17	6%	1	85%	99	15%	18	94%	60	6%	4	79%	54	21%	14	86%	56	14%	9	85%	63	15%	11
Liver	87%	121	13%	18	89%	16	11%	2	87%	102	13%	15	92%	59	8%	5	85%	58	15%	10	85%	55	15%	10	89%	66	11%	8
Lymph Nodes	92%	128	8%	11	89%	16	11%	2	92%	108	8%	9	92%	59	8%	5	91%	62	9%	6	91%	59	9%	6	93%	69	7%	5
Skin	92%	128	8%	11	94%	17	6%	1	91%	107	9%	10	94%	60	6%	4	90%	61	10%	7	89%	58	11%	7	95%	70	5%	4
Brain	94%	130	6%	9	94%	17	6%	1	93%	109	7%	8	95%	61	5%	3	93%	63	7%	5	92%	60	8%	5	95%	70	5%	4
Lung	94%	130	6%	9	83%	15	17%	3	95%	111	5%	6	97%	62	3%	2	91%	62	9%	6	92%	60	8%	5	95%	70	5%	4
Colon	97%	135	3%	4	100%	18	0%	0	97%	113	3%	4	97%	62	3%	2	97%	66	3%	2	95%	62	5%	3	99%	73	1%	1
Pleura	97%	135	3%	4	100%	18	0%	0	97%	113	3%	4	97%	62	3%	2	97%	66	3%	2	98%	64	2%	1	96%	71	4%	3
Bone Marrow	99%	138	1%	1	94%	17	6%	1	100%	117	0%	0	100%	64	0%	0	99%	67	1%	1	98%	64	2%	1	100%	74	0%	0
Spleen	99%	138	1%	1	100%	18	0%	0	99%	116	1%	1	100%	64	0%	0	99%	67	1%	1	100%	65	0%	0	99%	73	1%	1

	Overall				STS								17βHSD2								Aromatase							
					Negative				Positive				Negative				Positive				Arom 1-4				Arom 5-6			
	no metastasis, %, N		Metastasis %, N		no metastasis, %, N		Metastasis %, N		no metastasis, %, N		Metastasis %, N		no metastasis, %, N		Metastasis %, N		no metastasis, %, N		Metastasis %, N		no metastasis, %, N		Metastasis %, N		no metastasis, %, N		Metastasis %, N	
Bone	86%	119	14%	20	75%	43	25%	14	93%	75	7%	6	88%	22	12%	3	85%	96	15%	17	85%	34	15%	6	86%	85	14%	14
Liver	87%	121	13%	18	81%	46	19%	11	91%	74	9%	7	88%	22	12%	3	87%	98	13%	15	83%	33	18%	7	89%	88	11%	11
Lymph Nodes	92%	128	8%	11	84%	48	16%	9	98%	79	2%	2	96%	24	4%	1	91%	103	9%	10	95%	38	5%	2	91%	90	9%	9
Skin	92%	128	8%	11	88%	50	12%	7	96%	78	4%	3	100%	25	0%	0	91%	103	9%	10	95%	38	5%	2	91%	90	9%	9
Brain	94%	130	6%	9	91%	52	9%	5	95%	77	5%	4	96%	24	4%	1	93%	105	7%	8	95%	38	5%	2	93%	92	7%	7
Lung	94%	130	6%	9	88%	50	12%	7	98%	79	2%	2	96%	24	4%	1	93%	105	7%	8	90%	36	10%	4	95%	94	5%	5
Colon	97%	135	3%	4	98%	56	2%	1	98%	79	2%	2	100%	25	0%	0	97%	110	3%	3	98%	39	3%	1	97%	96	3%	3
Pleura	97%	135	3%	4	98%	56	2%	1	98%	79	2%	2	100%	25	0%	0	97%	110	3%	3	100%	40	0%	0	96%	95	4%	4
Bone Marrow	99%	138	1%	1	98%	56	2%	1	100%	81	0%	0	100%	25	0%	0	99%	112	1%	1	98%	39	3%	1	100%	99	0%	0
Spleen	99%	138	1%	1	100%	57	0%	0	99%	80	1%	1	100%	25	0%	0	99%	112	1%	1	98%	39	3%	1	100%	99	0%	0

Supplementary Table 2 Site of metastasis and expression of markers in the primary.
